# Co-Ultramicronized Palmitoylethanolamide/Luteolin-Induced Oligodendrocyte Precursor Cell Differentiation is Associated With Tyro3 Receptor Upregulation

**DOI:** 10.3389/fphar.2021.698133

**Published:** 2021-06-30

**Authors:** Laura Facci, Massimo Barbierato, Mariella Fusco, Pietro Giusti, Morena Zusso

**Affiliations:** ^1^ Department of Pharmaceutical and Pharmacological Sciences, University of Padua, Padua, Italy; ^2^ Scientific Information and Documentation Center, Epitech Group SpA, Padua, Italy; ^3^ IRCCS San Camillo Hospital, Venice, Italy

**Keywords:** oligodendrocyte progenitor cells, TAM receptors, MBP, CNPase, PeaLut

## Abstract

Remyelination in patients with multiple sclerosis frequently fails, especially in the chronic phase of the disease promoting axonal and neuronal degeneration and progressive disease disability. Drug-based therapies able to promote endogenous remyelination capability of oligodendrocytes are thus emerging as primary approaches to multiple sclerosis. We have recently reported that the co-ultramicronized composite of palmitoylethanolamide and the flavonoid luteolin (PEALut) promotes oligodendrocyte precursor cell (OPC) maturation without affecting proliferation. Since TAM receptor signaling has been reported to be important modulator of oligodendrocyte survival*,* we here evaluated the eventual involvement of TAM receptors in PEALut-induced OPC maturation. The mRNAs related to TAM receptors -Tyro3, Axl, and Mertk- were all present at day 2 *in vitro.* However, while Tyro3 gene expression significantly increased upon cell differentiation, Axl and Mertk did not change during the first week *in vitro*. Tyro3 gene expression developmental pattern resembled that of MBP myelin protein. In OPCs treated with PEALut the developmental increase of Tyro3 mRNA was significantly higher as compared to vehicle while was reduced gene expression related to Axl and Mertk. Rapamycin, an inhibitor of mTOR, prevented oligodendrocyte growth differentiation and myelination. PEALut, administered to the cultures 30 min after rapamycin, prevented the alteration of mRNA basal expression of the TAM receptors as well as the expression of myelin proteins MBP and CNPase. Altogether, data obtained confirm that PEALut promotes oligodendrocyte differentiation as shown by the increase of MBP and CNPase and Tyro3 mRNAs as well as CNPase and Tyro3 immunostainings. The finding that these effects are reduced when OPCs are exposed to rapamycin suggests an involvement of mTOR signaling in PEALut effects.

## Introduction

Multiple sclerosis (MS) is an inflammatory demyelinating disease of the central nervous system (CNS) characterized by axonal and neuronal degeneration. It mainly affects individuals in their early adult life, and has an enormous impact on body functions, quality of life and social costs that rise with increasing disability. MS prevalence worldwide is quite heterogeneous with high levels in North America and Europe (>100/100,000 inhabitants) and low rates in Eastern Asia and sub-Saharan Africa (2/100,000 population) (Leray et al., 2016).

MS is thought to be an immune-mediated disorder, in which the body’s immune system attacks CNS myelin causing alterations in the transmission of nerve signals between the brain and spinal cord and other parts of the body. The CNS myelinating cells are oligodendrocytes which are generated from oligodendrocyte progenitor cells (OPCs). Injuries to oligodendrocytes can be followed by a remyelination process leading to the formation of new myelin sheaths by newly formed oligodendrocytes (Franklin and Ffrench-Constant, 2008). Although remyelination can occur extensively in some people with MS (Patani et al., 2007), it often fails, especially in the chronic phase of the disease (Nakahara et al., 2009), promoting axonal and neuronal degeneration and progressive disease disability.

All the currently available therapies for MS are immunosuppressants and immunomodulators (Baecher-Allan et al., 2018). Despite their ability to ameliorate the length of relapses and the related symptoms, their side effects and inefficacy in counteracting the progression of the disease prompted to search for innovative strategies to recovery/improve neurological functions of patients. In this respect, drug-based therapies able to promote endogenous remyelination capability of oligodendrocytes are emerging as primary approaches to MS (Franklin and Ffrench-Constant, 2017). Within the context of these strategies, we have recently reported that the co-ultramicronized composite of palmitoylethanolamide and the flavonoid luteolin (PEALut) enhance morphological complexity and expression of both mRNA for the membrane-anchored myelin enzyme 2′,3′-cyclic nucleotide 3′-phosphodiesterase (CNPase) and myelin basic protein (MBP). The increase of MBP gene expression is associated with an increase of the corresponding protein (Barbierato et al., 2015; Skaper et al., 2018). PEALut has been shown to act as protective agent in different experimental models of CNS diseases (Caltagirone et al., 2016; Crupi et al., 2016; Siracusa et al., 2017). Its activity has been shown to be superior than ultramicronized-PEA alone (Cordaro et al., 2016). The active molecules of composite, ultramicronized palmitoylethanolamide and luteolin, have complementary and additive pharmacological activities, due to the ability to interact with different targets involved in the inflammatory response (Petrosino and Di Marzo, 2017; Ashaari et al., 2018). However, the mechanisms involved in PEALut-induced OPC maturation are not clarified.

Recent studies have shown that TAM receptors signaling is an important modulator of oligodendrocyte survival both *in vitro* and *in vivo* (Binder and Kilpatrick, 2009). They promote oligodendrogenesis, increase myelination by oligodendrocytes and their deficiency delays recovery following cuprizone-induced demyelination (Binder et al., 2011; Goudarzi et al., 2016).

All this evidence prompted us to examine the eventual involvement of TAM receptors in PEALut-induced OPC maturation. The mRNAs related to TAM receptors (Tyro3, Axl, and Merkt) and myelin proteins MBP and CNPase were analyzed during the progression of OPCs into a more differentiated phenotype in PEALut-treated cortical primary OPCs *vs* vehicle-treated OPCs. The mammalian target of rapamycin (mTOR) signaling is essential for oligodendrocyte differentiation (Tyler et al., 2009). mTOR is activated during OPC differentiation *in vivo* and *in vitro*, and its inhibition arrests oligodendrocyte differentiation at the late progenitor stage. For this reason, the expression of the TAM receptors and myelin proteins was also evaluated following exposition of OPCs to the rapamycin which inhibits some of the functions of mTOR.

## Materials and Methods

### Materials

Tissue culture media, N2 supplement, antibiotics and fetal bovine serum (FBS) were obtained from Thermo Fisher Scientific (Rodano, Italy); poly-D-lysine hydrobromide (mol wt 70,000–150,000), poly-L-lysine hydrobromide (mol wt 70,000–150,000), 3,3′ ,5-triiodo-L-thyronine, L-thyroxine, rapamycin, papain, DNase I (bovine pancreas), trypsin inhibitor, Pluronic F68 and all other biochemicals were purchased from Sigma-Aldrich (Milan, Italy) unless otherwise specified; Falcon tissue culture plasticware was purchased from BD Biosciences (SACCO srl, Cadorago (CO), Italy). Sterilin petri plastic dishes (10 cm Ø) were from Sarstedt (Verona, Italy). Co-ultramicronized PEALut (10:1 mass ratio) was kindly provided by Epitech Group SpA, Saccolongo (PD), Italy. Primary antibodies: rabbit anti-Tyro3 antibody (14H68L29, Thermo Fischer Scientific), mouse anti-CNPase antibody (C5922, Sigma-Aldrich); secondary antibodies: Alexa Fluor 488 goat anti-mouse and Alexa Fluor 555 goat anti-rabbit (Thermo Fischer Scientific).

### Oligodendrocyte Progenitor Cell Culture

Experiments were carried out following the National Institutes of Health guidelines for the care and use of laboratory animals and those of the Italian Ministry of Health (art. 31, D.L. 26/2014). The University of Padua’s Institutional Animal Care and Use Committee approved the experimental protocols used in this study (41451.N.N8P). Cultures of mixed glial cells were prepared from postnatal day 1 rat pup cortex as described (Rosin et al., 2004; Skaper et al., 2012). Briefly, tissue dissociates were plated in 75 cm^2^ poly-L-lysine-coated (10 μg/ml) tissue culture flasks at a density of 1.5 brains/flask and grown in high-glucose Dulbecco’s modified Eagle’s medium with 2 mM glutamine, 100 units/ml penicillin/50 μg/ml streptomycin, 50 μg/ml gentamicin, and 10% FBS (growth medium). Medium was changed after 24 h. Upon reaching confluence (approximately 7 days later), microglia were dislodged by shaking the flasks for 1 h at 200 rpm in a rotatory shaker (37°C) and the medium discarded. The flasks were re-fed with fresh growth medium and returned to the incubator for another 3 days. These flasks were subjected to a second cycle of shaking (6 h); the culture supernatant was subsequently transferred to plastic Petri dishes (Sterilin) and incubated for 25 min at 37°C (5% CO_2_/95% air) to allow differential adhesion of any remaining microglia.

The final cell suspension containing ∼95% oligodendrocytes (Rosin et al., 2004) was collected and centrifuged (200 g, 5 min). The resulting cell pellet was re-suspended in Sato medium (1 ml/well) (DMEM supplemented to contain 400 ng/ml 3,3',5-triiodo-L-thyronine, 400 ng/ml L-thyroxine, 2 mM L-glutamine, 50 U/ml penicillin, and 50 μg/ml streptomycin, 1× N2 supplement) and 0.5% (v/v) FBS and plated at 150,000 cells per well in a 24-well plate (unless otherwise indicated) coated with poly-D-lysine. The plating day was named day 1 *in vitro* (DIV 1).

### Oligodendrocyte Progenitor Cell Culture Treatments

#### Palmitoylethanolamide/Luteolin Suspension Preparation and Treatment

PEALut was prepared as a 5 mM stock solution in 10% (w/v) Pluronic F-68. PEALut suspension was sonicated for 20 min in a Elmasonic S (Singen, Germany) sonicating water bath, then diluted into the desired culture medium at 100× final concentration, and added (10 μl/ml) directly to the cell cultures without exchange of medium. The concentration of Pluronic F-68 was maintained constant at 0.02% in all cultures.

OPCs cultured in Sato medium were treated with PEALut (10 μM) or corresponding vehicle, either on the day of plating or on the following day. After different times of incubation, as indicated in each experiment, cells were processed for real-time polymerase chain reaction (PCR) analysis or immunocytochemistry.

#### Rapamycin Treatment

The compound was prepared as a 20 μM stock solution in dimethylsulfoxide (DMSO) and then diluted 100-fold in the desired culture medium (0.2 μM working solution, 1% DMSO). The day after plating (*i.e*., at DIV 2), the working solution was added to the cultures (10 μl/ml) to achieve a final concentration of 2 nM rapamycin. After 30 min incubation, PEALut or vehicle were added, as above. All cultures contained 0.02% Pluronic F-68 and 0.01% DMSO (final concentration).

### Real-Time Polymerase Chain Reaction

Total RNA was extracted from cells by QIAzol lysis reagent (Qiagen), according to the manufacturer’s instructions, at the times indicated in figure legends.

Reverse transcription was performed with SuperScript IV reverse transcriptase (Thermo Fisher Scientific). The real-time PCR reaction was performed as described previously (Contarini et al., 2019). Primer sequences were: βACT, 5′-GAT​CAG​CAA​GCA​GGA​GTA​CGA​TGA-3′, 5′-GGT​GTA​AAA​CGC​AGC​TCA​GTA​ACA-3'; MBP, 5′-TCC​GAG​GAG​AGT​GTG​GGT​TT-3′, 5′- TGGA​ACG​ATC​TGG​AGG​GTT​T-3′; Tyro3, 5′-ACT​ATT​ATC​GTC​AGG​GCT​GTG​C-3′, 5′-ACA​GTA​TAC​AAG​TTG​TCA​GCC​AA-3′; Axl, 5′-CCT​TCG​GTG​TGA​CAA​TGT​GGG-3′, 5′-GTC​GTA​AAT​CTC​ACT​GTT​CTC​CA-3′; Mertk, 5′-GGG​AAA​TAG​CAA​CAC​GGG​GAA​G-3′, 5′-GTG​GCC​GTG​GAG​AAG​GTA​ATC-3′; CNPase, 5′-GAC​CTG​GTC​AGC​TAT​TTT​GGC​A-3′, 5′-GCA​TAT​TCT​TCT​GCC​CCG​GTG-3′. Amounts of each gene product were calculated using linear regression analysis from standard curves, demonstrating amplification efficiencies ranging from 95 to 100%.

Dissociation curves were generated for each primer pair, showing single-product amplification. Data were normalized to β-actin mRNA level.

### Immunocytochemistry

OPCs were cultured on poly-D-lysine-coated 13 mm diameter cover glasses placed in a 24 multiwell plate. PEALut was added to a final concentration of 10 μM, after having allowed 1 h for cells to attach. Following 8 days of incubation, cells were fixed with 4% paraformaldehyde for 30 min at 4°C, washed 3 × 5 min with phosphate-buffered saline (PBS)/0.05% Triton X-100, and blocked with PBS/10% FBS for 1 h at room temperature.

The cells were then processed for immunostaining with anti-CNPase (1:100) and anti-Tyro3 (1:100) primary antibodies. Cells were then washed 3 × 5 min with PBS and incubated for 1 h at room temperature with Alexa Fluor 488 goat anti-mouse and Alexa Fluor 555 goat anti-rabbit secondary antibodies (1:1000), diluted in PBS. Nuclei were labeled with 4,6-diamidino-2-phenylindole (DAPI; 0.1 μg/ml). Cover glasses were mounted beneath glass slides using Fluoromount-G 14 (Southern Biotech, AL, United States ) to reduce the amount of fluorochrome quenching and photobleaching during fluorescence image acquisitions. Immunostaining controls included omission of the primary antibody to evaluate background signal. Images were acquired using a Leica DFC 480 digital camera, mounted on a Leica DMI4000 B fluorescence microscope (Leica Microsystems GmbH, Wetzlar, Germany) with a 10X objective. Microscope, lamp intensity, and camera settings were kept constant for all images. Images from 10 random fields of each condition were analyzed by ImageJ software (version 1.44p, NIH, MD, United States ), after subtracting background levels. Image acquisition and analysis were performed by an investigator blind to the experimental conditions.

### Statistics

Data are given as mean ± SEM (standard error of the mean). Statistical analysis to determine group differences were performed either by one- or two-way ANOVA analysis, followed by post hoc Holm-Sidak’s or Tukey’s tests for multiple comparisons. Significance was taken at *p*< 0.05. Statistical analysis was carried out using GraphPad Prism 6.0 (GraphPad Software, Inc., San Diego, CA, United States).

## Results

### Gene Expression of Tyro3, Axl, and Mertk Receptors in Differentiating Oligodendrocytes *in vitro*

The mRNA expression of Tyro3, Axl, and Mertk was evaluated in OPCs at different DIV in parallel with the mRNA expression of MBP and CNPase. Tyro3, Axl, and Mertk mRNAs were all expressed at DIV 2 as well as MBP and CNPase mRNAs. In later times (*i.e.*, at DIV 4 and 8), Tyro3 gene expression significantly increased over time with cell differentiation (*p*< 0.001; one-way ANOVA), likewise the genes codifying for myelin protein MBP (*p*< 0.005; one-way ANOVA). For both Tyro3 and MBP mRNAs the maximal increases were observed at DIV 4 (*p*= 0.001 and *p*= 0.0316 DIV 2 *vs* DIV 4*,* respectively; *post hoc* Tukey’s multiple comparison test).

In contrast, the expression of Axl, Mertk, and CNPase mRNAs did not change over time with cell differentiation (Table 1).

**TABLE 1:** Gene expression of TAM receptors (Tyro3, Axl, Mertk) and MBP and CNPase in oligodendrocyte precursors at different days *in vitro* (DIV).

	DIV			*p*	*p*	*p*
	2	4	8	2 *vs* 4	2 *vs* 8	4 *vs* 8
Tyro3	1.0 ± 0.0	5.3 ± 0.5	5.1 ± 0.5	0.0010	0.0012	ns
Axl	1.0 ± 0.0	1.2 ± 0.2	1.0 ± 0.2	ns	ns	ns
Mertk	1.0 ± 0.0	0.8 ± 0.1	0.9 ± 0.1	ns	ns	ns
MBP	1.0 ± 0.1	8.1 ± 0.5	10.5 ± 3.0	0.0316	0.0048	ns
CNPase	1.0 ± 0.0	1.6 ± 0.1	1.5 ± 0.3	ns	ns	ns

Data obtained from two separated experiments (6 values) are expressed as mean ± SEM. Data were analyzed with one-way ANOVA followed by *post hoc* Tukey’s multiple comparison test. *p* values refer to *post hoc* analysis.

### PEALut Induced mRNA Expression of Tyro3 Genes in Differentiating OPCs Likewise MBP and CNPase mRNAs

**FIGURE 1:**
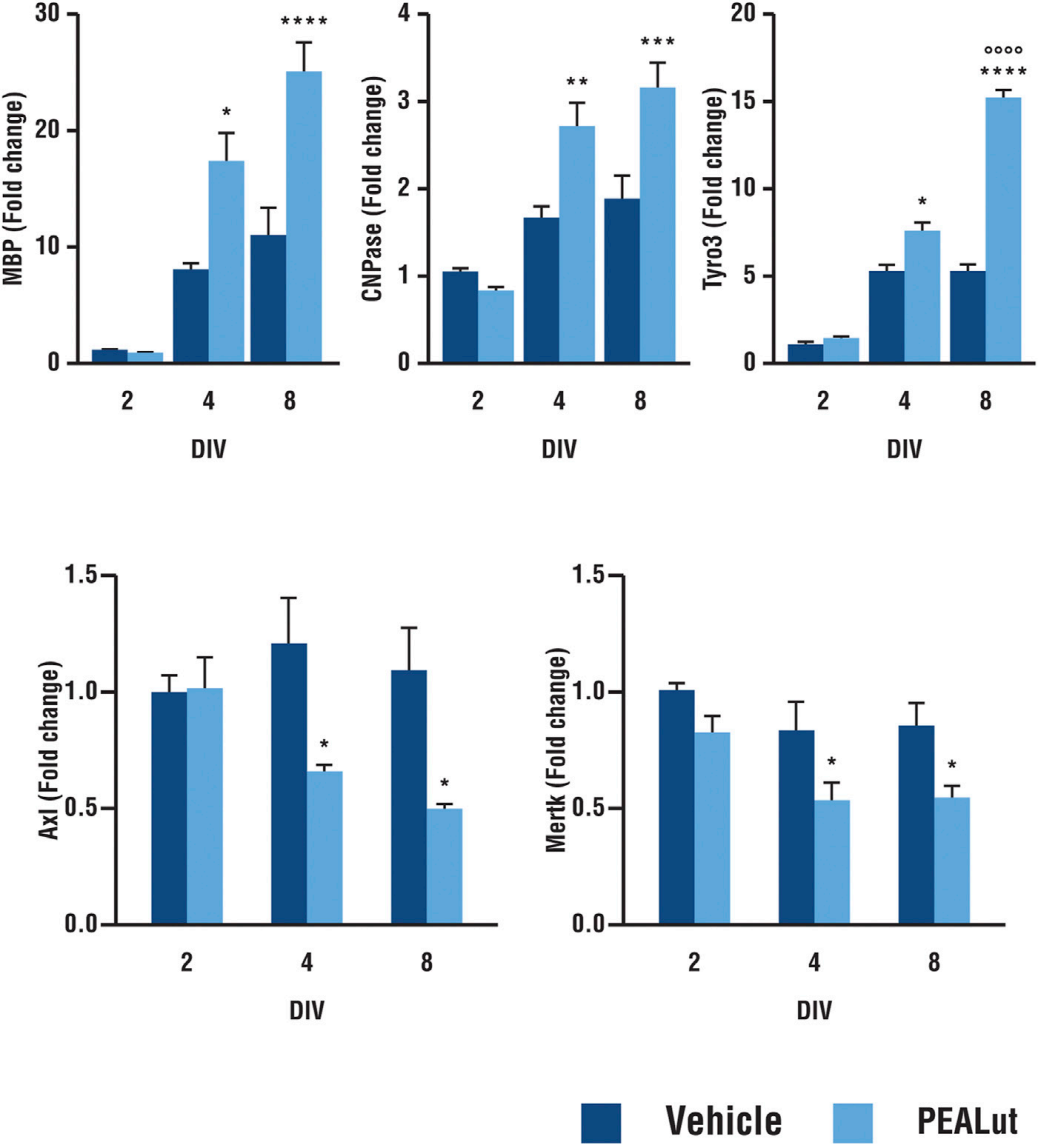
Time-dependent increase in expression of MBP, CNPase, Tyro3, Axl and Mertk mRNAs in PEALut-treated OPCs. PEALut time-dependently increases expression of MBP, CNPase and Tyro3 in differentiating OPCs, while it induces a reduction of Axl and Mertk mRNAs. Cultures of OPCs were treated on the plating day (DIV 1) with 10 μM PEALut and processed at DIV 2, 4, and 8 for real-time PCR. Data from two separated experiments (6 values) are expressed as fold-increase with respect to the control (vehicle) at DIV 2 and are means ± SEM. Data were analyzed with two-way ANOVA followed by *post hoc* Holm-Sidak’s multiple comparison test. MBP: *p < 0.05 *vs* Vehicle; ****p < 0.0001 *vs* Vehicle. CNPase: **p < 0.005 *vs* Vehicle; ***p < 0.001 *vs* Vehicle; Tyro3: *p < 0.05 *vs* Vehicle; ****p < 0.0001 *vs* Vehicle; °°°°*p* < 0.0001 *vs* PEALut 4 days old culture. Axl and Mertk: *p < 0.05 *vs* Vehicle.

Tyro3 mRNA expression was evaluated in PEALut-treated OPC cells (10 μM, once, the day of plating, *i.e*., DIV 1) in parallel with MBP and CNPase mRNAs. Tyro3 mRNA in differentiating oligodendrocyte was significantly higher in OPCs treated with 10 µM PEALut. Tyro3 gene expression, compared to DIV 2, increased by 7.5 ± 0.7 and 15.3 ± 0.3 at DIV 4 and 8, respectively. PEALut-induced Tyro3 mRNA increase was evident at DIV 4 (*p*< 0.05 *vs* corresponding vehicle), with a further increase at DIV 8 (*p*< 0.0001 *vs* corresponding vehicle as well as *vs* PEALut DIV 4; Figure 1, upper panel). The incubation of OPCs with PEALut, as expected (Barbierato et al., 2015), led to a time-dependent up-regulation of MBP and CNPase mRNA levels. In OPCs at DIV 4, MBP mRNA was approximately doubled in cells treated with 10 µM PEALut as compared to those treated with vehicle (*p*< 0.05, Figure 1, upper panel). A further MBP mRNA increase occurred in PEALut treated OPCs at DIV 8 (Figure 1, upper panel). An increase of gene expression was also observed for myelin protein CNPase; this increase reached the maximal value at DIV 4 (*p*< 0.001) without a further increase at DIV 8 (Figure 1, upper panel).

### PEALut Induced a Decrease in the Expression of Axl and Mertk in Differentiating OPCs

Treatment of differentiating OPCs with 10 µM PEALut induced a small but consistent downregulation of Axl and Mertk mRNA levels. When Axl and Mertk mRNA expression was evaluated at different times in culture (Figure 1, bottom panel), the reduction of gene expression was statistically significant at both DIV 4 and 8 (*p*< 0.05 *vs* vehicle).

### PEALut Prevented the Rapamycin-Induced Decrease in Tyro3, MBP, and CNPase mRNA Expression in OPCs at DIV 8

**FIGURE 2:**
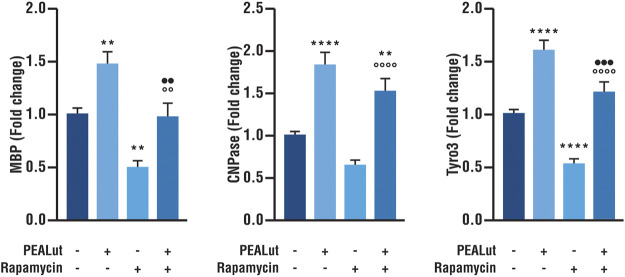
PEALut effects on rapamycin-treated OPCs at DIV 8. Cultures of OPCs were treated with 10 μM PEALut at DIV 2 and processed at DIV 8 for real-time PCR. Rapamycin was added 30 min before PEALut. Data are expressed as mean ± SEM. Data were analyzed with one-way ANOVA followed by *post hoc* Holm-Sidak’s multiple comparisons test. Data were obtained from five separated experiments (15 values). MBP: **p < 0.005 *vs* vehicle; °°*p* < 0.005 *vs* rapamycin; ••*p* < 0.005 *vs* PEALut. CNPase: **p < 0.01 *vs* vehicle; ****p < 0.0001 *vs* vehicle; °°°°*p* < 0.0001 *vs* rapamycin; Tyro3: ****p < 0.0001 *vs* vehicle; °°°°*p* < 0.0001 *vs* rapamycin; •••*p* < 0.001 *vs* PEALut.

The activation of mTOR is essential for oligodendrocyte differentiation (Wahl et al., 2014). As a consequence, in line with our previous findings (Barbierato et al., 2015) the addition of the potent and selective inhibitor of mTOR rapamycin to cell cultures induced a significant reduction in the gene expression of myelin proteins MBP and CNPase in OPCs at DIV 8 (Figure 2). Furthermore, rapamycin-treated OPCs showed also a decreased Tyro3 mRNA expression as compared to vehicle (Figure 2). PEALut significantly prevented the decreased Tyro3, MBP and CNPase mRNAs expression induced by rapamycin (Figure 2), so that gene expression in PEALut group was similar to that of the vehicle.

### PEALut Induced a Tyro3 and CNPase Protein Upregulation in OPCs at DIV 8

**FIGURE 3:**
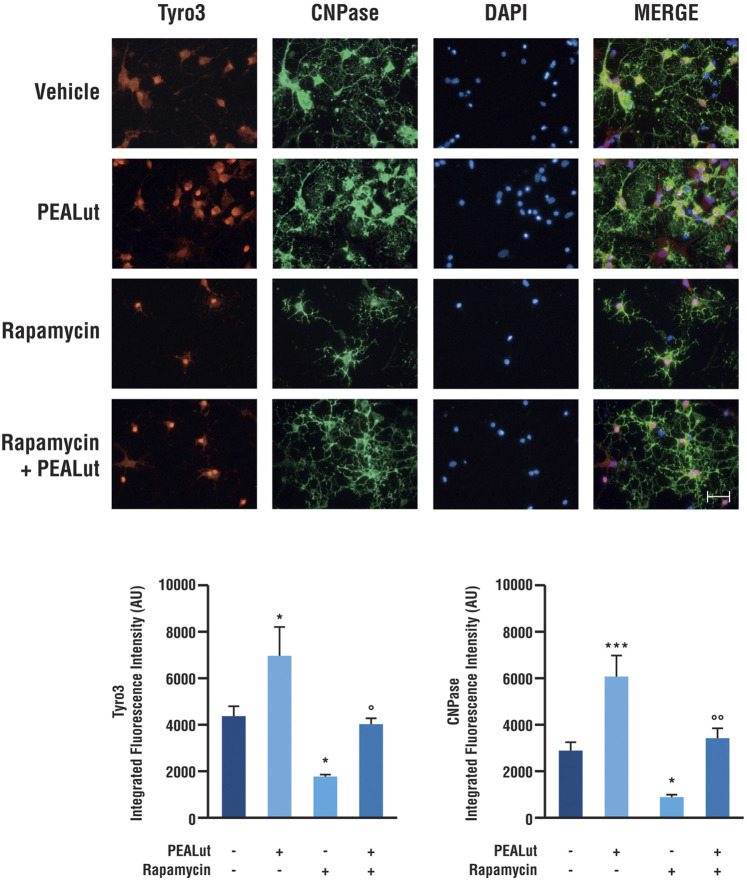
Tyro3 and CNPase immunostaining in OPCs at DIV 8. Cultures of OPCs were treated PEALut at DIV 2 and processed at DIV 8 for immunofluorescence analysis. Rapamycin was added 30 min before PEALut. Scale bar, 20 μm. Graphs show the quantitative analysis of the integrated intensity of fluorescence. Data are expressed as mean ± SEM. Tyro3: *p < 0.05 *vs* Vehicle; °*p* < 0.05 *vs* PEALut. CNPase: *p < 0.05 *vs* Vehicle; ***p < 0.001 *vs* Vehicle; °°*p* < 0.005 *vs* PEALut.

To evaluate whether the variations of Tyro3 gene expression were accompanied by variations of the corresponding protein, OPCs exposed to rapamycin and treated with PEALut or corresponding vehicle were processed for immunostaining at DIV 8. Anti-Tyro3 primary antibody was used to visualize Tyro3 protein. In parallel, the primary antibody anti-CNPase was used to visualize the CNPase structural protein primarily expressed in the myelinating oligodendrocytes. Rapamycin significantly induced a decrease in both Tyro3 and CNPase immunostaining (Figure 3). In OPCs exposed to rapamycin and treated with PEALut, Tyro3 and CNPase immunofluorescences were comparable to those of vehicles (Figure 3). At the same time, naïve OPCs treated with PEALut showed a significant increase in both CNPase and Tyro3 immunostaining (Figure 3); however, the total cell number did not increase compared to vehicle, as demonstrated by cell nuclei counts (data not shown).

The immunofluorescence quantitative analysis confirmed a significant increase in both CNPase (*p*< 0.0001) and Tyro3 (*p*< 0.05) protein levels in OPCs treated with PEALut. On the contrary, rapamycin exposure induced a decrease of both CNPase (*p* < 0.0001) and Tyro3 (*p*< 0.05) protein levels in OPCs. Once again, PEALut was able to reverse the effect of rapamycin (Figure 3).

### PEALut Prevented the Rapamycin-Induced Increases in Axl and Mertk mRNA Expression in OPCs at DIV 8

**FIGURE 4:**
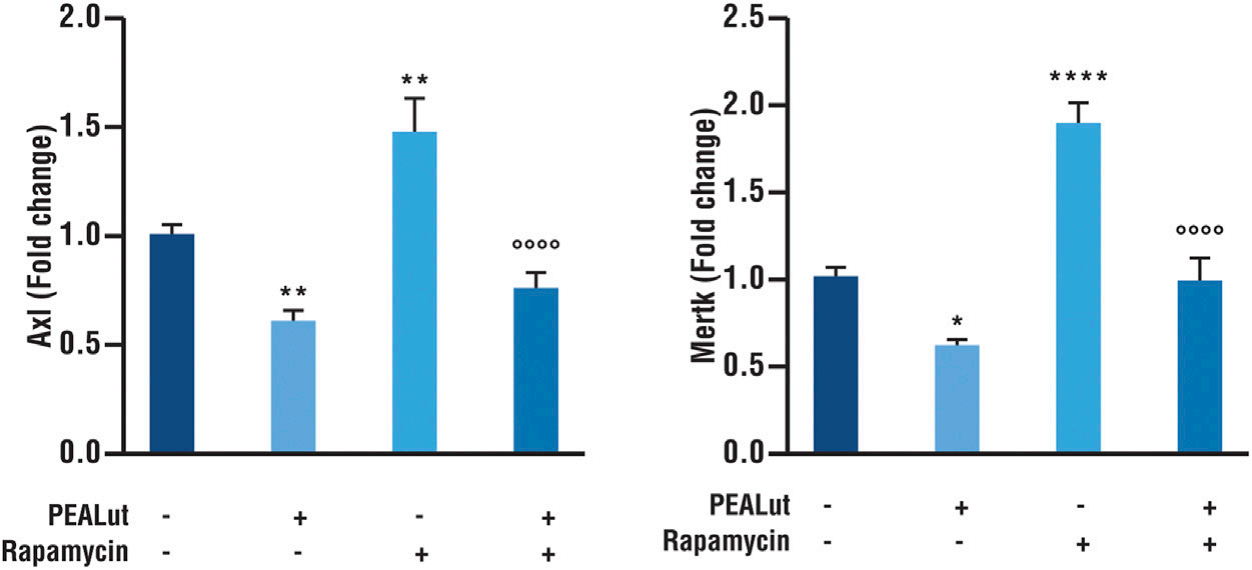
PEALut reversed the rapamycin-induced increases in Axl and Mertk mRNA in OPCs at DIV 8. Cultures of OPCs were treated with 10 μM PEALut at DIV 2 in cultures and processed at DIV 8 for real-time PCR. Rapamycin was added 30 min before PEALut. Data are expressed as mean ± SEM. Data were analyzed with one-way ANOVA followed by post hoc Holm-Sidak’s multiple comparison test. Data were obtained from four separated experiments (12 values) Axl: **p< 0.005 *vs* Vehicle; °°°°*p*< 0.0001 *vs* Rapamycin. Mertk: *p< 0.05 *vs* Vehicle; ****p< 0.0001 *vs* Vehicle; °°°°*p*< 0.0001 *vs* Rapamycin.

The evaluation of Axl and Mertk mRNAs was performed in OPCs at DIV 8 exposed to rapamycin and treated with PEALut or vehicles. PEALut treatment to naive OPCs down regulated both Axl and Mertk mRNAs as compared to corresponding vehicles (*p* < 0.005 and *p*< 0.05 *vs* vehicle, respectively; Figure 4). The exposure of OPCs to rapamycin significantly increased the expression of both Axl and Mertk mRNAs as compared to corresponding vehicles (*p*< 0.05 and *p*< 0.0001 *vs* vehicle, respectively; Figure 4). PEALut treatment prevented the rapamycin-induced increases of both Axl and Mertk mRNAs expression to corresponding vehicle (vehicle *vs* PEALut/Rapamycin).

## Discussion

TAM receptors Tyro3, Axl and Mertk have been reported to be coexpressed in myelinating stage oligodendrocytes. Tyro3 is highly expressed and widely distributed in the CNS, its expression is maximal during late postnatal development and in the adult (Prieto et al., 2000). TAM receptors are activated by the binding of two closely-related ligands, Growth Arrest Specific Gene 6 (Gas6) and Protein S (ProS) (Binder and Kilpatrick, 2009). Gas6 has been shown to be a pro-survival factor for human oligodendrocytes, and Gas6 protein has been shown to be upregulated in the CSF of patients with a chronic inflammatory demyelinating disease (Binder and Kilpatrick, 2009).

We herein report that all three genes are expressed at detectable levels in OPCs obtained from newborn rats at postnatal day 1. However, Tyro3 mRNA levels are higher as compared to Axl and Mertk mRNAs. Moreover, the expression of Tyro3 rises with increasing days in culture while no relevant changes are observed in the Axl and Mertk mRNAs. This evidence is in line with findings showing that Axl and Mertk are expressed at lower levels throughout development, and do not exhibit a postnatal increase in expression (Prieto et al., 2000).

Changes in Tyro3 expression during DIV are analogous to those of MBP, a gene coding for a protein essential for oligodendrocyte morphogenesis at late stages of cell differentiation and whose role in the process of myelination of nerves in the nervous system is well known (Galiano et al., 2006). Analogy in the development of the two genes supports the hypothesis of a role of Tyro3 in the processes of cellular maturation and myelination.

Myelination process occurs relatively late in development in a defined temporal sequence (Snaidero and Simons, 2014). For example, in the mouse spinal cord, it begins at birth and is nearly completed by the 60th postnatal day in most brain regions (Baumann et al., 2001). Tyro3 receptor has been reported to be an important regulator of CNS myelination (Akkermann et al., 2017), since it influences developmental myelination by regulating myelin thickness in CNS (Akkermann et al., 2017) and promotes Schwann cell myelination in the peripheral nervous system (Miyamoto et al., 2015); therefore, its expression is expected to increase over time, such as happened with MBP (Barbierato et al., 2015; Skaper et al., 2018).

In the CNS, Tyro3 has been reported to regulate the nature of myelin repair during and after exposure to cuprizone, a copper chelator used to obtain a toxic demyelination. Moreover, the loss of Tyro3 led to a delayed myelination and reduced myelin thickness, both *in vitro* and *in vivo*. This effect was caused by an impaired myelin production potentially related to the oligodendrocyte Tyro3 protein (Akkermann et al., 2017). These findings suggest that restoring the expression of Tyro3 mRNA alongside with the level of corresponding protein could increase myelination processes when Tyro3 signaling is dysregulated. In this contest, the ability of PEALut to increase mRNA expression of Tyro3 makes it as a valuable candidate for Tyro3 signaling recovery. This result supports the role of PEALut in the promotion of myelination and suggest a Tyro3 involvement in PEALut activity.

Furthermore, in our *in vitro* system, we demonstrated that rapamycin, an inhibitor of mTOR, inhibits the increase of Tyro3 mRNA and its corresponding protein, after PEALut treatment. Likewise, rapamycin inhibits the developmental increase of MBP mRNA as well as the appearance of mature oligodendrocyte phenotypes. These findings further support a role of Tyro3 in oligodendrocyte differentiation and myelination. Rapamycin is an immunosuppressant drug therefore can inhibit mTOR protein preventing oligodendrocyte growth differentiation and myelination (Norrmén and Suter, 2013; Barbierato et al., 2015). Previously, we had already shown that rapamycin attenuates morphological maturation, reduces protein content, and induces a decrease in myelin-related gene expression, including MBP in OPCs after 4 days of treatment (Barbierato et al., 2015), an effect counteracted by PEALut treatment. All together, this evidence suggests that, as in the neurons (Pierce and Keating, 2014) and in the Schwann cells (Miyamoto et al., 2015), also in oligodengrocytes progenitors, Tyro3 receptor is able to activate their differentiation and myelination through the involvement of the PI3K/AKT/mTOR cascade (Wood et al., 2015). In addition, at least in the latter PEALut could be able to promote this pathway (Barbierato et al., 2015).

In contrast to Tyro3, Axl and Mertk receptors are poorly expressed both in CNS and OPCs (Ray et al., 2017); their physiological role is not completely known, but normally they do not participate in the maturation/ differentiation processes of OPCs (O'Guin et al., 2014), therefore their expression does not increase over time (Prieto et al., 2000). In our cell cultures, Axl and Mertk mRNAs were detectable in culture at all analyzed days, however their expression was lower as compared to Tyro3 and a significant decrease was observed with OPC maturation. In the CNS, Axl and Mertk are mainly expressed in microglia, and their loss leads to enhanced inflammation in the CNS and delayed removal of myelin debris in experimental models of MS (Ray et al., 2017; O'Guin et al., 2014). Axl expression is elevated in astrocytes and oligodendrocytes in chronic active and chronic silent MS lesions (Weinger et al., 2011). These data suggest that Axl and Mertk play important signaling roles in multiple cell types within the CNS during episodes of inflammation and repair. It is likely that in CNS oligodendrocytes collaborate with microglia and astrocyte to clear myelin debris and to promote phagocytosis as well as autophagy as do Schwann cells in peripheral nervous system. The down regulation of Axl and Mertk induced by PEALut might facilitate the recovery of physiological oligodendrocytes functions. A PEALut treatment to naïve OPCs induced a significant reduction both in Axl and Mertk transcrips. The reduced expression of the two genes after PEALut treatment is compatible with the morphological change of the OPCs and the shift into a more differentiated cellular phenotype. The finding further supports the role of PEALut in promoting OPC morphological maturation likely through Tyro3 receptor. In contrast, rapamycin that prevents OPC cell growth, induces a significant increase in Axl and Mertk mRNAs expression. Interestingly, at DIV 8 PEALut was able to counteract the rapamycin-induced increase in Axl and Mertk mRNA expression by restoring the expression levels of the two receptors to the basal values.

In our study we observed that the expression of CNPase, in contrast to MBP, did not increase with OPC differentiation during the first week *in vitro*. It has been reported that as oligodendrocytes differentiate *in vitro* they begin to produce large membranous expansions from their cell bodies and along the lengths of cell processes. MBP and CNPase have all been identified immunochemically as components of these *in vitro* membrane expansions, CNPase appearing in membrane sheets some days before MBP (Knapp et al., 1988). In neonatal mouse brain cell cultures, the maximal number of cells expressing CNPase and MBP was observed at DIV 18, however the increase of cells expressing CNPase in the culture was observed starting from DIV 10 while no variations were observed at previous times in culture (Amur-Umarjee et al., 1990). A similar developmental pattern for CNPase and MBP has been reported in primary cultures of fetal rat brain: CNPase activity began to increase by DIV 8–10 and reached their maxima by DIV 20. However, different environmental signals (cellular and/or humoral) regulate the onset of the gene expressions relevant to CNPase activity and MBP accumulation (Pfeiffer et al., 1981). Our observation on CNPase and MBP expression in the first week of cultured OPCs are substantially in line with these previous studies. Moreover, we report that PEALut is able to promote myelination and differentiation on increasing the expression of both proteins, while maintaining their balance not only in naïve cultures but also following exposure to rapamycin.

## Conclusion

In conclusion, in OPC cultures derived from rat pup cortex, during the first week *in vitro* development pattern of Tyro3 is different from Axl and Mertk. The expression of Tyro3 increases with OPC differentiation while Axl and Merk do the opposite. We here confirm that PeaLut promotes oligodendrocyte differentiation as shown by the increase of MBP and CNPase mRNAs, an effect accompanied by TAM receptor Tyro3 enhancement. PEALut-induced increase of Tyro3, MBP, and CNPase mRNA expression is significantly reduced when OPCs are exposed to rapamycin, thus suggesting an involvement of mTOR signaling in PEALut effects.

PEALut has been shown to act as protective agent in different experimental models of CNS diseases (Contarini et al., 2019). The active molecules of composite, palmitoylethanolamide and luteolin, interact with different cellular and molecular targets allowing to achieve multiple effects. PEA is endowed with important neuroprotective, anti-inflammatory, and analgesic actions. The peroxisome proliferator-activated receptor (PPAR)-α is the molecular target that directly mediates some of the neuroprotective, anti-inflammatory, and analgesic effects of PEA (Contarini et al., 2019). Indirect mechanisms of action for PEA have also demonstrated that PEA potentiates anandamide actions at cannabinoid receptors (“entourage” effects). In line with our results, it has been reported that cannabinoid receptor agonists modulate oligodendrocyte differentiation by activating PI3K/Akt and mTOR pathways (Gomez et al., 2011). Noteworthy, also the activation of Tyro 3 receptor by Gas6 upregulates components of transcriptional and translational machinery that involves PI3K/Akt and mTOR pathways to promote synaptic plasticity (Prieto et al., 2000). Lut is a widespread flavone known to have antioxidant and cytoprotective properties (Contarini et al., 2019). Importantly, Lut has been shown to improve the morphology of PEA: naïve PEA shows a morphology characterized by large flat crystals, and the presence of very small quantities of Lut stabilizes the microparticles inhibiting PEA crystallization process, thus improving the pharmacological PEA properties (Adami et al., 2019).

Altogether, the data obtained support the hypothesis that PEALut may act via a mTOR-dependent molecular pathway, however, further studies will be needed to elucidate this possibility.

## Data Availability

The raw data supporting the conclusions of this article will be made available by the authors, without undue reservation.
